# Fixed versus flexible gonadotropin releasing hormone antagonist protocol in women with polycystic ovary syndrome undergoing in vitro fertilization: An RCT

**DOI:** 10.18502/ijrm.v22i8.17230

**Published:** 2024-10-14

**Authors:** Hanieh Fatehi, Robab Davar, Elham Nikfarjam, Fatemeh Bayati

**Affiliations:** ^1^Research and Clinical Center for Infertility, Yazd Reproductive Sciences Institute, Shahid Sadoughi University of Medical Sciences, Yazd, Iran.; ^2^Gynecology and Infertility Department, Shiraz Fertility Center, Shiraz, Iran.; ^3^Department of Obstetrics and Gynecology, Clinical Research Development Unit, Hajar Hospital, Shahrekord University of Medical Sciences, Shahrekord, Iran.

**Keywords:** Gonadotropin-releasing hormone, Polycystic ovary syndrome, Fertilization in vitro, Oocytes.

## Abstract

**Background:**

Despite the extensive use of the gonadotropin-releasing hormone (GnRH) antagonist protocol in treating infertile women, particularly those with polycystic ovary syndrome (PCOS), there have not been sufficient evidence to compare the flexible and fixed variants in in vitro fertilization (IVF) cycles.

**Objective:**

This study aims to assess the treatment outcomes of flexible and fixed types of GnRH-antagonist protocol for IVF in women with PCOS.

**Materials and Methods:**

In this randomized clinical trial, 150 infertile women with PCOS, who were candidates for IVF, and referred to the Yazd Research and Clinical Center for Infertility, Yazd, Iran between October 2023 and February 2024 were included. Participants were divided into 2 groups (n = 75/each) based on the type of antagonist protocol (fixed or flexible). GnRH antagonist administration started on the 5 ${\;}^{\text{th}}$ day of gonadotropin treatment in the fixed group. In the flexible group when there was at least one follicle 12–14 mm, GnRH antagonist was started. Finally, the number of metaphase II oocyte, the quality of embryos, the duration of the stimulation cycle, the dose of gonadotropin, the number of GnRH-antagonist, and the rate of ovarian hyperstimulation syndrome were evaluated.

**Results:**

No statistically significant difference was observed in terms of cycle length and the total dose of gonadotropin between groups. Nevertheless, a notable distinction was observed in the total number of oocytes (17.84 vs. 15.5, p = 0.023) and mature oocytes (13.64 vs. 11.83, p = 0.019) in the flexible group compared to the fixed group.

**Conclusion:**

In conclusion, the IVF outcomes are more favorable in women with PCOS undergoing the flexible GnRH-antagonist protocol compared to the fixed protocol.

## 1. Introduction

Polycystic ovary syndrome (PCOS) is a prevalent endocrine disorder, affecting 5–18% of women in their reproductive years. It is unique that it lacks specific signs or symptoms, instead characterized by disruptions in follicular development that lead to chronic anovulation-a leading cause of anovulatory infertility in 80% of affected women (1, 2). For women with infertility due to PCOS who do not respond to controlled ovarian stimulation (COS) methods like clomiphene and letrozole, or those with other infertility causes, assisted reproductive technology (ART) offers a beneficial alternative (1). However, these women often exhibit increased sensitivity to ovarian stimulation due to higher antral follicle count and anti-Müllerian hormone (AMH) levels, which can result in ovarian hyperstimulation syndrome (OHSS). Thus, selecting the most suitable ovarian stimulation protocol and recognizing PCOS risk factors is essential (3).

Research extensively supports the use of gonadotropin-releasing hormone (GnRH) antagonists to reduce OHSS occurrence in PCOS participants. Consequently, European society of human reproduction and embryology guidelines recommend the antagonist protocol for this group (3). The GnRH-antagonist protocol has become increasingly popular in ART cycles due to its ability to competitively bind to GnRH receptors in the pituitary, rapidly inhibiting gonadotropin release and shortening treatment duration (4). The antagonist protocol is favored because it inhibits the luteinizing hormone surge in the COS cycle without the adverse effects of hypoestrogenism or prolonged downregulation induced by GnRH agonists. Studies have demonstrated that it achieves live birth rates (LBRs) comparable to GnRH-agonist protocols while reducing gonadotropin consumption (5, 6), shortening ovarian stimulation periods, and lowering OHSS risk (7, 8).

Antagonist protocols are differentiated by their initiation times: fixed protocols start antagonist administration on days 5–6 of ovarian stimulation, whereas flexible protocols begin when the dominant follicle reaches 14 mm. For women with PCOS, the flexible antagonist protocol may be more effective due to small antral follicles' sensitivity to exogenous follicle-stimulating hormone; however, limited studies have compared these 2 protocols (9). Moreover, meta-analysis results indicate that a fixed regimen without pretreatment may lead to higher ongoing pregnancy rates (OPR) than other regimens for individuals with a normal ovarian response. However, there is a lack of evidence supporting this approach for poor or hyper-responders; more research is needed (10).

In fact, the gap of knowledge primarily revolves around the nuanced response of women with PCOS to different antagonist protocols in ART cycles. While extensive research supports the use of GnRH antagonists to decrease OHSS occurrence, there is a lack of definitive evidence on the efficacy of fixed vs. flexible antagonist protocols specifically in women with PCOS who are hyper responders to ovarian stimulation. Moreover, there are certain limitations, such as the challenge of applying findings from a population with a normal ovarian response to those with PCOS. There's also a need for a deeper understanding of how different initiation times and dosages in antagonist protocols affect the hormonal balance in PCOS cases.

These gaps highlight the necessity for further research to refine ART protocols tailored to this specific group, ensuring treatments are both personalized and effective. In light of these considerations, our study examined 2 types of antagonist protocols including fixed and flexible in infertile women with PCOS.

## 2. Materials and Methods

### Study design and participants

In this RCT, 150 infertile women with PCOS who referred to the Research and Clinical Center for Infertility, Yazd, Iran from October 2023 to February 2024 for in vitro fertilization (IVF) treatment were randomly enrolled into 2 groups (n = 75/each) based on the type of antagonist protocol used (either fixed or flexible). The inclusion criteria included women diagnosed with PCOS according to the Rotterdam criteria (11) aged between 20 and 40 yr, and with a body mass index (BMI) of 20–30. Women with severe endometriosis, pelvic neoplasia, and severe male factor infertility (including severe oligozoospermia, cryptozoospermia, or an absence of spermatozoa in the ejaculate) were excluded.

### Sample size

With considering the average of 15 metaphase II (MII) oocytes in the fixed group and the average of 18 in the flexible group, and considering the standard deviation of 6.5, with a confidence level of 95% and a test power of 80%, the sample size was calculated using the following formula (9). The participant count was approximately 75 in each group. The sample size calculation was performed using PASS15 software.


$$n=\frac{{\left(Z_{1-\frac{\propto }{2}}\pm Z_{1-\beta }\right)}^{2}\times {2S}^{2}}{{\left(\mu_{1}-\mu_{2}\right)}^{2}}$$

### COS

In all participants, 150 IU of recombinant follicle-stimulating hormone (Cinnal-F, Cinna Gen Co., Iran) was administered starting from the second day of the menstrual cycle. Subsequently, in the fixed group, GnRH-antagonist administration began on the 5 ${\;}^{\text{th}}$ day of gonadotropin treatment. In the flexible group, GnRH-antagonist administration commenced when there was at least one follicle measuring 12–14 mm. The transvaginal ultrasound protocol for follicular monitoring was performed by an infertility fellowship researcher on the 2 ${\;}^{\text{nd}}$ day of the menstrual cycle, and gonadotropin treatment was initiated. Then, on the 7 ${\;}^{\text{th}}$ day of the cycle, another ultrasound is conducted to measure the size of the follicles and assess the response to treatment. Depending on the ovarian response to stimulation, ultrasounds are performed every 2–3 days to measure the size of the follicles and determine the day for trigger administration. Additionally, a daily dose of 0.25 mg of Cetrorelix acetate (Cetronax, Ronak Darou Co., Iran) was continued until the trigger day in both groups. In both groups, ultrasound monitoring was used to track follicle development. When there were at least 2 follicles measuring ≥ 17 mm in diameter, triggering was performed. The trigger involved administering 1500–5000 IU of human chorionic gonadotropin (Folgnan, Darou Pakhsh, Iran) along with either a GnRH agonist, 1 mg of Buserelin Acetate (CinnaFact, Cinna Gen, Tehran, Iran) or GnRH agonist alone (if there was a risk of OHSS). The human chorionic gonadotropin dosage was determined based on the amount of estradiol and the count of follicles in the trigger day.

Approximately 34–36 hr after the trigger, ovarian puncture was carried out under general anesthesia and ultrasound guidance. Following this, 1–2 cleavage-stage embryos were transferred (embryos with grade A, B, and C), while the remaining embryos were frozen. Estradiol and progesterone levels were measured on the trigger day. If there was a risk of OHSS or if the serum progesterone level exceeded 2.25 ng/ml, all embryos were frozen (2). OHSS is defined as having a follicle count of ≥ 25 or a serum estradiol level of ≥ 4000. Furthermore, OHSS is divided into mild, moderate, severe, and critical groups based on the severity of symptoms (12).

An experienced embryologist evaluated the quality of all usable embryos on the 3 ${\;}^{\text{rd}}$ day of development in culture. This assessment followed the morphological criteria that established previously (13). Each embryo was assigned a letter grade (A, B, C, or D) based on its appearance. Here is what each grade represents:

• Grade A: Excellent quality embryos with no fragmentation and uniform, similar-sized blastomeres (cells).

• Grade B: Good quality embryos with minimal fragmentation ( < 20%) and uniform, similar-sized blastomeres.

• Grade C: Fair-quality embryos with moderate fragmentation (20–50%) and blastomeres of varying sizes.

• Grade D: Poor quality embryos with significant fragmentation ( > 50%) and blastomeres of varying sizes. These embryos were not used for transfer due to lower implantation potential.

### Randomization

In this study, we employed the `Random Allocation1' software for the randomization of participants. The process was based on the simple randomization method, and it was carried out by a statistician who generated a list for the allocation of samples to the 2 study groups.

### Maintaining concealment and blinding

A sealed envelope method was used to preserve the concealment of the randomization sequence. An equal number of sealed envelopes to the sample size were prepared, each containing the designation of the intervention group. Moreover, blinding was not possible due to the method of intervention, hence, it was not performed.

### Study variable and outcomes 

Demographic variables of participants such as age, duration of infertility, and type of infertility were recorded based on participant's history. Moreover, BMI as a person's weight in kilograms divided by the square of height in meters and AMH based on blood test, which corresponds to participant ovarian reserve was determined. After an ovarian puncture, the number of MII oocytes as the primary outcome was manually counted by the embryologist. Whereas the embryo quality as the first secondary outcome was assessed by microscopic manual counting 1–2 days after the ovarian puncture. Furthermore, the duration of the stimulation cycle, which refers to the length of time from the start of COS until the trigger for oocyte maturation was measured. Moreover, a dose of gonadotropin was administered during COS to stimulate follicle growth. The number of antagonist doses administered was recorded as the number of GnRH antagonists and the rate of OHSS, which is a potential complication of COS was assessed 3–7 days after the trigger using physical examination and ultrasound.

### Ethical considerations

This study was approved by the Ethics Committee of Yazd Reproductive Sciences Institute, Yazd, Iran (Code: IR.SSU.RSI.REC.1402.013). Following registration with the Iranian Registry of Clinical Trials on October 9, 2023, which was updated on May 12, 2024, participant enrollment began on October 12, 2023. The purpose of this project, along with its pros and cons, was initially explained to the participants, and written informed consent was obtained from all participants before enrollment in the trial.

### Statistical analysis

We used the IBM SPSS Statistics (Statistical Package for the Social Sciences, version 26, Chicago, IL, USA) for statistical analysis. Also, the means ± standard deviations were used for presentation of continuous variables, while frequencies (%) for categorical data. Furthermore, the Mann-Whitney or *t* test were used for comparing continuous variables between research groups and the Chi-square test for the categorical variables. A 2-tailed p-value < 0.05 was considered for the statistically significant differences in each test.

## 3. Results

**Figure 1:**
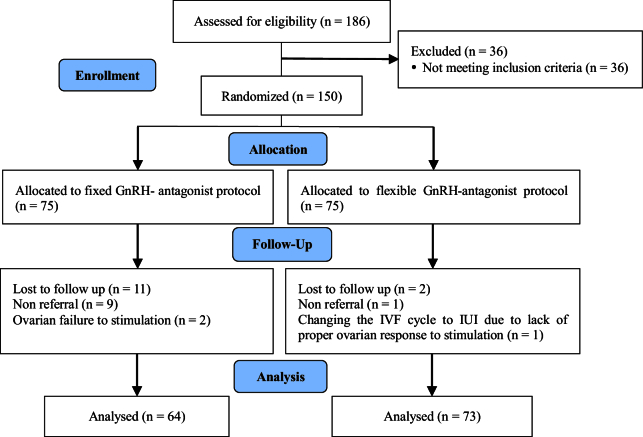
CONSORT flowchart of the research plan. GnRH: Gonadotropin-releasing hormone, IVF: In vitro fertilization, IUI: Intra-uterine insemination.

Based on the results, 186 women met the criteria for evaluation. 16 were excluded due to severe male factor infertility and 20 were excluded due to BMI outside the specified range. 150 women were randomly assigned to both fixed and flexible groups (n = 75/each). In the fixed group, 9 women did not return for treatment and 2 women had their cycle canceled due to poor ovarian response to stimulation. In the flexible group, 1 woman did not return for treatment, and in one woman, the IVF cycle was converted to intra-uterine insemination. Finally, 64 women in the fixed group and 73 women in the flexible group were analyzed (Figure 1).

Table I displays the baseline characteristics of participants in both fixed and flexible groups. Notably, no statistically significant differences were observed between groups regarding age, BMI, infertility duration, infertility type, and AMH levels.

The number of oocytes obtained in the flexible group was significantly higher compared to the fixed group (17.84 vs. 15.5, p = 0.023). The number of MII oocytes in the flexible group was significantly higher compared to the fixed group (13.64 vs. 11.83, p = 0.019). The number of injected cetrotide vials in the flexible group was significantly less compared to the fixed group (4.45 vs. 6.28, p = 0.000). Furthermore, no significant differences were observed between the 2 groups in the case of the duration of the ovulation stimulation cycle, the amount of gonadotropin consumed, and the embryo grade, as well as the number of 2PN embryos. No significant difference was observed between groups in terms of prevalence and OHSS severity (Table II).

**Table 1:** Comparison of the baseline characteristics of participants in the flexible vs. fixed group


**Variables**		**Fixed (n = 64)**	**Flexible (n = 73)**	**P-value**
Age (yr)*		30.91 ± 5.61	30.40 ± 4.96	0.470
Duration of infertility (yr)**		6.37 ± 4.14 (5, 5)	5.90 ± 3.76 (5, 5)	0.562
Type of infertility***				
	**Primary**	52 (81.2)	55 (75.3)	
	**Secondary**	12 (18.8)	18 (24.7)	0.404
BMI (Kg/m^2^)*		26.72 ± 3.10	27.04 ± 3.93		0.127
AMH (ng/ml)**		6.38 ± 4.56 (5.1, 5.4)	5.56 ± 2.95 (4.8, 3.95)	0.715
*Data presented as Mean ± SD, Student's *t* test. **Data presented as Mean ± SD (Median, interquartile range), Mann-Whitney Test, ***Data presented as n (%), Chi-square. BMI: Body mass index, AMH: Anti-Mullerian hormone				

**Table 2:** Comparison of COS outcomes in fixed/flexible groups


**Variables**		**Fixed (n = 64)**	**Flexible (n = 73)**	**P-value**
Cycle duration (days)*		11.44 ± 2.03 (11, 2.75)	11.29 ± 1.31 (11, 2)	0.923
Gonadotropin injections (dosage)*		1598.25 ± 301.5 (1500, 450)	1617 ± 317.25 (1500, 150)	0.624
Cetrotide injections (n)*		6.28 ± 2.14 (6, 3)	4.45 ± 2.40 (4, 2)	< 0.001
Oocytes (n)*		15.5 ± 6.91 (15, 8)	17.84 ± 6.75 (17, 8)	0.023
Oocytes in metaphase II (n)*		11.83 ± 6.33 (11, 6.75)	13.64 ± 5.39 (13, 7)	0.019
Oocytes in metaphase I (n)*		1.33 ± 1.85 (1, 2)	1.21 ± 1.65 (1, 2)	0.750
Germinal vesicle (n)*		2.14 ± 2.41 (1, 3)	2.30 ± 2.92 (1, 3)	0.914
2PN (n)*		7.75 ± 5.29 (7, 5.75)	8.09 ± 4.63 (7, 6.5)	0.441
Embryo grading*				
	**A**	1.28 ± 1.56 (1, 2)	1.08 ± 1.56 (0, 2)	0.450
	**B**	3.78 ± 2.86 (3.5, 3)	3.70 ± 2.68 (3, 3)	0.735
	**C**	1.83 ± 1.786 (1, 1.75)	2.31 ± 2.05 (2, 2)	0.040
	**D**	0.033 ± 0.18 (0, 0)	0.23 ± 0.84 (0, 0)	0.122
Incidence rate of OHSS**				
	**Mild**	37 (57.8)	44 (60.3)	
	**Moderate**	26 (40.6)	28 (38.4)	
	**Severe**	1 (1.6)	1 (1.4)	
	**Critical**	0 (0)	0 (0)	0.868
*Data presented as Mean ± SD (Median, interquartile range), Mann-Whitney test. **Data presented as n (%), Chi-square. COS: Controlled ovarian stimulation, 2PN: 2 pronuclei, OHSS: Ovarian hyperstimulation syndrome				

## 4. Discussion

This clinical research is an RCT that studies the clinical outcome of the fixed GnRH-antagonist protocol in COS for IVF in women with PCOS, with the flexible protocol. This study revealed a significantly higher number of total oocytes retrieved (p = 0.023) in the flexible group (17.84) compared to the fixed group (15.5). This difference may be due to the antagonist in the flexible group being prescribed when the follicles are larger, leading to a greater number of mature follicles and, ultimately, an increased number of oocytes retrieved. Additionally, the flexible group required significantly fewer antagonist injections due to the later start of the antagonist. Furthermore, no significant differences were observed between the 2 protocols in terms of embryo grade, cycle duration, gonadotropin consumption, and OHSS incidence. Over the years, researchers have compared various GnRH-antagonist protocols in IVF to identify the optimal approach. A meta-analysis compared fixed and flexible GnRH-antagonist protocols in IVF. No significant difference was observed in pregnancy rates between the 2 groups (14). Also, a direct comparison between the fixed vs. flexible GnRH-antagonist protocols in IVF was followed by the 4 new RCTs to refine the optimal approach (15–18). Moreover, a meta-analysis reviewed and compared the 7 RCTs in women undergoing ART with fixed and flexible protocols and found that the differences between the 2 groups are not significant for the length of the cycle, which is in accordance with our finding. Furthermore, the fixed protocol showed a lower number of cumulus-oocyte complexes and a lower level of estradiol on the trigger day. Although less antagonist is used in the flexible protocol, recent analysis showed that the OPR is lower in this protocol (19). Furthermore, in a retrospective study conducted in China on women of advanced maternal age, researchers found no significant difference between the fixed and flexible GnRH-antagonist groups in terms of cumulative LBRs and time to live birth. Importantly, the timing of GnRH-antagonist initiation in advanced maternal aged women did not significantly affect their long-term pregnancy outcome, including cumulative LBRs and time to live birth (20).

Although a similar study was previously conducted, some of its findings align with the current study, while others contradict it (5). This study revealed a significantly higher number of total oocytes retrieved (p = 0.023) in the flexible group (17.84) compared to the fixed group (15.5). In agreement with our finding, they reported that the number of oocytes retrieved was significantly higher in flexible (14.75) compared to fixed protocol (6.9) (p < 0.01) (5). However, the total and mature MII oocytes in the flexible group of the mentioned study were considerably lower than those obtained by our study. In comparison with our findings and as per another study, the longer duration of infertility in flexible group was the reason for the lower values of total and mature MII oocytes (21). In addition, the same trend was observed for mature MII oocytes in both studies with p = 0.019 and p < 0.01, respectively, and no significant differences were observed between the 2 protocols in terms of embryo grade. However, it is interesting that the increase in the abovementioned outcomes in flexible group compared with fixed group was more than 100% in a previous study (5) and this increase was only about 15% for our study. Therefore, the flexible antagonist protocol resulted in more number of good quality oocytes and embryos and more over improved the possibility for cryopreservation for future cycles for PCOS infertile women.

However, it is vital to consider the diverse results by others, whereas for the participants with predicted high ovary response except PCOS, they found that there is no difference in total number of oocytes retrieved between the fixed and flexible protocol (9). It appears as a distinct type of high ovarian responders, PCOS participants typically exhibit lower follicular sensitivity to follicle-stimulating hormonecompared to normal ovarian responders and other high ovarian responders. However, due to inappropriate ovarian stimulation by exogenous follicle-stimulating hormone, these participants are prone to either a slow ovarian response or hyperstimulation. The heterogeneity among PCOS participants further increase the likelihood of unpredictable follicle development (9). In contrary and in agreement with previous studies, our findings showed that in flexible regime the gonadotropin injection was higher, but no significant difference in total dose of gonadotropin was observed (5, 22). Moreover, significantly fewer cetrotide injections were obtained for flexible group compared with fixed group, whereas no significant differences were observed between the 2 protocols in terms of cetrotide injections in the same study (5).

In fact, the flexible protocol for administering cetrotide, as highlighted in the recent study, offers significant advantages over traditional methods. One of the primary benefits is the reduced number of cetrotide vials required. This reduction not only lowers the overall cost of the treatment, making it more economically feasible for participants, but also enhances the practicality of the protocol. Fewer injections mean less discomfort and inconvenience for participants, which can improve adherence to the treatment regimen and overall participant satisfaction. Participants often find frequent injections burdensome, and minimizing this aspect can lead to a more positive treatment experience. This improvement in participant experience is crucial, as it can influence the psychological well-being of participants, which is an important factor in the overall success of fertility treatments. In conclusion, the flexible protocol for cetrotide administration presents a compelling case for its adoption in clinical practice. By reducing the number of injections, it addresses both economic and practical concerns, ultimately benefiting participants and enhancing the overall treatment experience.

### Strengths and limitations

The strength of this study was that few RCTs have been performed in this field in PCOS women. Moreover, the number of injectable steroid ampoules were less, which is better for the participant, is economical and cost-effective. Whereas a limitation of this study was that it focused on women with PCOS. Since PCOS women often have a higher risk of OHSS, most embryos were frozen for future cycles. This limited the ability to examine long-term pregnancy outcomes (clinical pregnancy, OPR, LBRs) within the study timeframe.

## 5. Conclusion

While the fixed GnRH-antagonist protocol is simpler and requires less monitoring, the flexible protocol is preferable for women with PCOS undergoing IVF. The flexibility of this protocol, based on the number of retrieved oocytes and the higher quality of mature oocytes, may lead to better outcomes and the possibility of cryopreservation for future cycles. The flexible approach holds promise for PCOS women in IVF and could potentially become the optimal protocol. However, further large-scale RCTs are needed to confirm this benefit and evaluate long-term pregnancy outcomes for a more definitive choice.

##  Data availability 

The authors confirm that the derived data supporting the findings of this paper are available from the corresponding author upon reasonable request.

##  Author contributions

H. Fatehi, R. Davar, and F. Bayati designed the study and conducted the research. H. Fatehi authored the paper and had access to all study data. E. Nikfarjam monitored and evaluated the result of the study. Further, R. Davar and E. Nikfarjam reviewed the article. All authors approved the final manuscript and are responsible for maintaining the integrity of the data and ensuring the accuracy of the presented information.

##  Conflict of Interest 

The authors declare that there is no conflict of interest.
